# Reliability and Agreement Assessment of Sarcopenia Diagnosis through Comparison of Bioelectrical Impedance Analysis and Dual-Energy X-ray Absorptiometry

**DOI:** 10.3390/diagnostics14090899

**Published:** 2024-04-25

**Authors:** Jung Hun Lee, Hee Jin Kim, Sanghun Han, Seong Jun Park, Myongheon Sim, Kang Hyun Lee

**Affiliations:** 1 Department of Emergency Medicine, Yonsei University Wonju College of Medicine, Wonju 26426, Republic of Korea; leejhuns@yonsei.ac.kr (J.H.L.); hj.kim@yonsei.ac.kr (H.J.K.); 2 MEDIANA Co., Ltd., Wonju 26365, Republic of Korea; windhan@mediana.co.kr (S.H.); sjpark@mediana.co.kr (S.J.P.); bmesim@mediana.co.kr (M.S.)

**Keywords:** sarcopenia, bioelectrical impedance analysis, dual-energy X-ray absorptiometry

## Abstract

A unified diagnostic criterion has yet to be established for sarcopenia. Therefore, we analyzed the reliability and validity of sarcopenia diagnosis using bioelectrical impedance analysis (BIA) compared with the gold standard, dual-energy X-ray absorptiometry (DEXA), and evaluated the predictive accuracy of BIA for diagnosis. The clinical trial, involving a total of 239 participants, was conducted between December 2018 and September 2019 on healthy volunteers without significant medical histories. The participants underwent health assessments, followed by sequential DEXA and BIA measurements. In both the low and normal appendicular skeletal muscle (ASM) groups, there were significant differences in the right arm, left arm, right leg, left leg, ASM, and ASM index (ASMI) between DEXA and BIA across all age groups (*p* < 0.05). BIA tended to overestimate compared to DEXA, but ASMI values for males and females were consistent with the criteria for sarcopenia. Bland–Altman analysis showed that each segment in both the low and normal ASM groups fell within the limits of agreement (LOA). The diagnosis of sarcopenia using BIA was significantly different from that using DEXA. However, it exhibited a significantly high correlation, fell within the LOA, and demonstrated high predictive accuracy. BIA can be considered an effective tool for diagnosing sarcopenia.

## 1. Introduction

Sarcopenia is characterized by a degenerative loss of skeletal muscle mass, quality, and strength associated with the aging process. The sarcopenia incidence rate has shown an annual increase of approximately 0.8% among individuals aged 50 years and above. Its prevalence rate is rapidly increasing in response to an ongoing global aging trend [1]. Additionally, the prevention and management of sarcopenia are emerging as significant clinical issues in public health [2,3,4,5]. According to domestic research, a meta-analysis targeting individuals aged 65 years and older revealed that 13.1% were diagnosed with sarcopenia, with males accounting for 14.9% and females for 11.4% [2]. Sarcopenia can lead to a decline in physical activity, difficulties in maintaining daily life functions, an increased risk of falls and fractures, a higher likelihood of entering long-term care facilities, and increased mortality rates. Additionally, there is an elevated risk of infection, an increase in the incidence of metabolic syndromes, such as cardiovascular diseases, diabetes, and hypertension; and an increase in the occurrence of circulatory system disorders [6].

Despite the severity of this disease, no single set of diagnostic criteria has yet been established [2]. Therefore, there has been a global increase in the volume of research studies on sarcopenia [7]. Since the establishment of diagnostic criteria by the European Working Group on Sarcopenia in Older Persons (EWGSOP) in 2010, which include low muscle mass and impaired muscle function (either reduced strength and/or diminished physical performance), the International Working Group on Sarcopenia, the Foundation for the National Institutes of Health, and the Asian Working Group for Sarcopenia (AWGS) have introduced comparable definitions with varying thresholds [8,9]. In 2016, sarcopenia was recognized in the International Classification of Diseases, Tenth Revision, Clinical Modification, under the code M62.84. In Korea, diagnostic codes for sarcopenia were included in the eighth revision of the Korean Standard Classification of Diseases in 2021 [2].

Sarcopenia has a higher prevalence in older populations; the decline in muscle mass usually begins at approximately the age of 40 years and onward [10]. The adverse effects of sarcopenia on both quality of life and healthcare demand can affect individuals in both middle-aged and older age groups [10,11]. Assessment of body composition is an important aspect of clinical assessment, and tracking changes in body composition over time can be an informative means of assessing the disease state [12]. Current methods for assessing body composition include dual-energy X-ray absorptiometry (DEXA), magnetic resonance imaging (MRI), and computed tomography (CT). Nevertheless, these approaches have drawbacks, including the need for a trained medical professional for measurements, associated costs, inconvenience, exposure to ionizing radiation, and difficulty in measuring body composition in daily life [12,13].

Recently, bioelectrical impedance analysis (BIA) devices for assessing body composition have become available on the market and are being utilized at various sites related to health and physical fitness care, as well as at home. BIA has progressed by including multiple frequencies and impedance measurements, resulting in improved accuracy and reliability when estimating body composition parameters, such as the percentage of body fat (BF), fat mass (FM), fat-free mass (FFM), and total body water (TBW) [14]. Compared with other methods, BIA offers simplicity, cost-effectiveness, rapidity, and noninvasiveness [13,14,15].

In this study, we aimed to evaluate the reliability and validity of sarcopenia diagnosis using BIA compared with the gold standard, DEXA, and to assess the predictive accuracy of BIA for diagnosis. We compared appendicular skeletal muscle mass (ASM), a diagnostic factor for sarcopenia, between BIA and DEXA, and evaluated the muscle masses of the arms and legs separately, which were used to calculate ASM. We further investigated the diagnostic accuracy across age groups and sexes.

## 2. Material and Methods

### 2.1. Sarcopenia

In 2014, the AWGS introduced a diagnostic algorithm for sarcopenia that considers characteristics specific to the Asian population, such as ethnicity, body size, lifestyle, and cultural background. This algorithm closely resembles that proposed by the EWGSOP. In 2019, the AWGS revised its consensus on both the diagnosis and treatment of sarcopenia [16,17,18]. Recognizing the challenges of assessing muscle mass in community settings, the AWGS endorsed the use of BIA to measure ASM in 2014. In 2019, the AWGS proposed additional strategies to identify individuals at risk for sarcopenia early, aiming to enable necessary interventions in settings without advanced diagnostic equipment [19]. For case findings, it is recommended to utilize either the calf circumference; the Strength, Assistance in walking, Rise from a chair, Climb stairs (SARC), and Falls scale; or the SARC and calf circumference.

Sarcopenia was evaluated by the following criteria: (1) muscle strength was defined as handgrip strength (males: <28 kg; females: <18 kg); (2) physical performance was assessed by the 6-meter walk (<1.0 m/s), 5-time chair stand test (≥12 s), or a Short Physical Performance Battery (≤9); and (3) ASM was defined with DEXA (males: <7.0 kg/m^2^; females: <5.4 kg/m^2^) or BIA (males: <7.0 kg/m^2^; females: <5.7 kg/m^2^). Sarcopenia is characterized by low ASM + low muscle strength or physical performance, and severe sarcopenia is characterized by low ASM + low muscle strength and physical performance. ASM was defined as the sum of the muscle masses of the four limbs, and the ASM index (ASMI) was calculated as ASM/height^2^ (kg/m^2^).

### 2.2. Study Design and Population

We used data collected from the recruitment of clinical trial participants at Wonju Severance Christian Hospital between December 2018 and September 2019. This clinical trial aimed to validate the accuracy and reliability of the body composition analyzer. The evaluation of BIA involved comparing its measurement results with those of DEXA and the TBW calculated using isotope dilution. We analyzed the collected data based on the criteria for sarcopenia.

The criteria for participant selection included healthy individuals without significant medical histories that could affect the clinical trial and voluntary participation. For children and adolescents under the age of 19 years, the trial proceeded with the consent of the legal guardians, typically parents, who provided their signatures. The exclusion criteria included pregnancy; the presence of metallic materials, such as pacemakers and stents; and insertion of implanted medical devices. Participants with infectious diseases or wounds on their palms, feet, or soles were also excluded.

The participants were provided with a comprehensive explanation of the purpose and procedures of the clinical trial before obtaining their consent. Additionally, participants were instructed to abstain from high-intensity exercise and alcohol consumption for 48 h prior to participating in the clinical trial to ensure they obtained sufficient sleep. Furthermore, eating, drinking, and running were prohibited 12 h before the measurement. Participants who consented to the clinical trial removed all metal items, such as rings, necklaces, bracelets, anklets; emptied their bladders; and changed into lightweight clothing. Subsequently, the participants’ health information was collected, followed by approximately 30 min of sufficient rest in a separate room before performing DEXA and BIA measurements.

This study was approved by the Institutional Review Board of Wonju College of Medicine, Yonsei University (CR217013), and a clinical trial was conducted in compliance with the International Organization for Standardization 14155 [20].

In this study, participants were divided into four groups according to age: youth (<19 years), younger (19 to 39 years), middle-aged (40 to 59 years), and elderly (≥60 years).

### 2.3. Measurement Procedures

#### 2.3.1. Anthropometric Survey

Height was measured using a stadiometer, and weight was measured using a scale. Additionally, the arm, abdominal, hip, and thigh circumferences were measured using a tape measure. All measurements were performed twice, and if the second measurement differed by more than 1% from the first, a repeat measurement was performed.

#### 2.3.2. DEXA

DEXA (Horizen W; Hologic Inc., Marlborough, MA, USA) was used to measure whole and regional body compositions with FM, lean soft tissue mass, and muscle mass. The participants underwent measurements under the guidance of medical professionals responsible for conducting DEXA scans within the hospital. The participants lay on the scanning table in the supine position, and they were instructed to ensure that their arms and legs were positioned accurately. During the measurement, participants were instructed to refrain from talking and moving. The DEXA was calibrated and managed according to hospital management procedures.

In this study, the ASM using DEXA was calculated as the sum of lean mass in the right arm (RA), left arm (LA), right leg (RL), and left leg (LL). Therefore, the calculated ASM was classified into low and normal ASM groups based on the criteria for sarcopenia diagnosis by DEXA.

#### 2.3.3. BIA

The i Series (Mediana Co., Ltd., Wonju, Korea) was used for BIA. The i Series was used to perform the multifrequency hand-to-foot BIA using the tetrapolar 8-point tactile electrode method. The low-end BIA module of the i Series uses multi-frequencies of 1–250 kHz, and the high-end BIA module uses those in the 1–1000 kHz range. Multifrequency was used to measure the impedance of each segment, such as the RA, LA, trunk, RL, and LL, and to predict several variables, including FM, %BF, FFM, lean mass, and TBW, through the measured impedance. The electrodes were cleaned, and the participants’ hands and feet were wiped with an antibacterial tissue before measurement. The hand electrodes were held in an upright position with the feet centered on the electrodes, and the arms were spread sufficiently wide to avoid contact between the arms and the torso. During the measurement, participants were instructed to refrain from talking and to maintain their position.

### 2.4. Statistical Analysis

Participant characteristics were presented using descriptive statistics, such as frequency distribution, mean, and standard deviation. The statistical analysis method considered the sample size, and parametric and non-parametric tests were used depending on normality. Comparisons of the characteristics between DEXA and BIA were conducted using the paired *t*-test or Wilcoxon signed-rank test. Age group comparisons between the low and normal ASM groups were performed using the independent t-test or Mann–Whitney U test. Relative agreement was analyzed by linear regression analysis, and the Pearson’s or Spearman’s correlation coefficient (r) was calculated. Bland–Altman analyses were used to compare the agreement between measurements. Limits of agreement (LOA) between methods were defined as the mean difference ±1.96 standard deviation (95% LOA). A confusion matrix was used for prediction evaluation. Statistical analyses were performed using the IBM SPSS Statistics 27 (IBM Corp., Armonk, NY, USA) and MATLAB R2023b (MathWorks, Natick, MA, USA).

## 3. Results

### 3.1. Study Characteristics

The clinical trial recruited a total of 239 participants. We compared the population characteristics between the low and normal ASM groups (Table 1). Participants were categorized into the following age groups: youth (36; 15.1%), younger (101; 42.3%), middle-aged (84; 35.1%), and elderly (18; 7.5%). The proportions of participants with low ASM were as follows: youth (25; 69.4%), younger (41; 40.6%), middle-aged (43; 51.2%), and elderly (8; 44.4%). The proportions of participants with normal ASM were as follows: youth (11; 9.0%), younger (60; 49.2%), middle-aged (41; 33.6%), and elderly (10; 8.2%).

In the comparison of males and females using DEXA and BIA, FM and %BF were higher in females (*p* < 0.05), whereas FFM and lean mass were higher in males (*p* < 0.05). When comparing the low and normal ASM groups, most of the FM, %BF, FFM, and lean mass values were higher in the normal ASM group (*p* < 0.05). In the total cohort and low and normal ASM groups, BIA measurements resulted in an underestimation of FM and %BF while overestimating FFM and lean mass (*p* < 0.05). In addition, there was a significant correlation between DEXA and BIA in all participants (FM, r^2^ = 0.939, *p* < 0.001; %BF, r^2^ = 0.902, *p* < 0.001; FFM, r^2^ = 0.984, *p* < 0.001; and lean mass, r^2^ = 0.983, *p* < 0.001). Anthropometric measurements revealed that in the total cohort and low and normal ASM groups, males had higher values for RA circumference (RAC), LA circumference (LAC), and waist circumference (WC) than females did. In the RAC, LAC, WC, hip circumference (HC), RL circumference (RLC), and LL circumference (LLC) groups, the normal ASM group showed higher values than those of the low ASM group (*p* < 0.05).

### 3.2. Differences in Measurement Data between the Two Devices

We present the DEXA and BIA results for the low and normal ASM groups, based on each segmental measurement and age, in Table 2 and Table 3. In all age groups with low and normal ASM, there were statistically significant differences between DEXA and BIA for the RA, LA, RL, LL, ASM, and ASMI (*p* < 0.05). In particular, BIA showed a tendency to overestimate values compared to DEXA, but the ASMI for males and females was 7.0 (0.9) kg/m^2^ and 5.7 (0.5) kg/m^2^, respectively, which is consistent with the sarcopenia diagnostic criteria of 7.0 kg/m^2^ for males and 5.7 kg/m^2^ for females. The difference in ASMI (BIA–DEXA) between age groups for low ASM was as follows: in males, the differences for the total cohort and youth, younger, middle-aged, and elderly groups were 0.8 (0.3), 0.7 (0.3), 0.9 (0.3), 0.9 (0.3), and 0.7 (0.3) kg/m^2^, respectively. For females, the differences were 0.8 (0.3), 0.8 (0.3), 0.8 (0.3), 0.9 (0.3), and 0.8 (0.1) kg/m^2^, respectively. In the normal ASM group, the differences for males were 0.8 (0.4), 0.9 (0.3), 0.8 (0.4), 0.9 (0.3), and 0.6 (0.1) kg/m^2^, respectively, and for females, they were 0.6 (0.3), 0.7 (0.2), 0.6 (0.3), 0.6 (0.3), and 0.5 (0.3) kg/m^2^, respectively.

### 3.3. Comparison of Reliability of Two Devices

The correlations between BIA and DEXA in the low-ASM group for the RL, LA, RL, LL, ASM, and ASMI were high, with values of 0.950, 0.948, 0.969, 0.968, 0.977, and 0.936, respectively. Similarly, in the normal ASM group, the correlations were high, with values of 0.949, 0.933, 0.974, 0.980, 0.980, 0.980, and 0.937, respectively (*p* < 0.05). Furthermore, the correlation of ASMI in the low ASM group was 0.925, 0.931, 0.915, and 0.975 for the youth, younger, middle-aged, and elderly groups, respectively. In the normal ASM group, the correlation coefficients were 0.891, 0.951, 0.895, and 0.980, respectively. RA, LA, RL, LL, ASM, and ASMI were highly correlated with sex. However, in some subgroups of the youth and elderly groups, statistical significance was not observed owing to lack of data.

### 3.4. Bland–Altman Analysis

**Figure 1:**
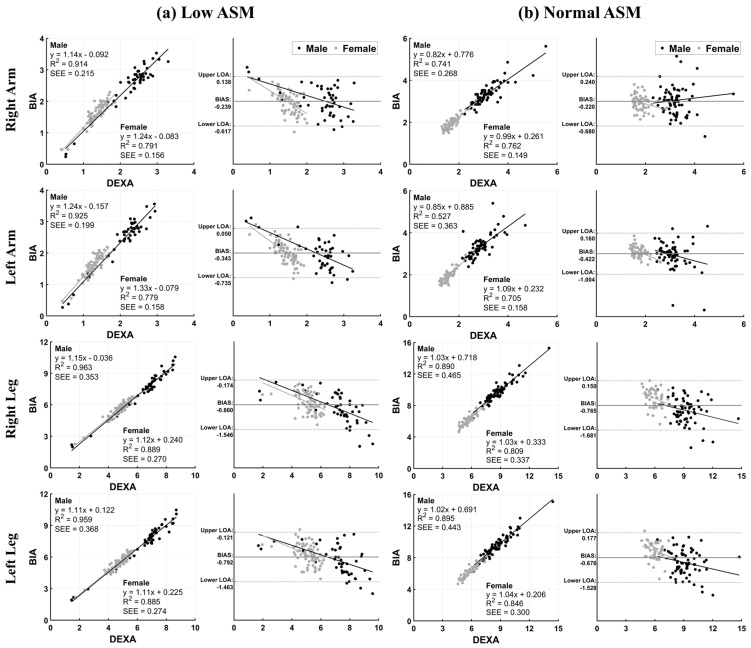
Linear regression and Bland–Altman analysis for all participants. The *x*-axis of the Bland–Altman analysis represents the average of the two measurements ([DEXA + BIA]/2), and the *y*-axis represents the difference between the two measurements (DEXA-BIA). ASM, appendicular skeletal muscle mass; DEXA, dual-energy X-ray absorptiometry; LOA, limits of agreement; SEE, standard error of the estimate.

We evaluated the measurement results of DEXA and BIA by segment using linear regression and Bland–Altman analysis. The r-squared values for the linear regression equations were high for the RA, LA, RL, and LL for all participants. In the low ASM group, the r-squared values were 0.914, 0.925, 0.963, and 0.959, respectively, for males and 0.791, 0.779, 0.889, and 0.885, respectively, for females (*p* < 0.05). In the normal ASM group, the r-squared values were 0.741, 0.527, 0.890, and 0.895, respectively, for males and 0.762, 0.705, 0.809, and 0.846, respectively, for females (*p* < 0.05). In the Bland–Altman analysis, each segment of the low and normal ASM groups was within the LOA. However, there was a tendency for systematic bias, with BIA results being overestimated, and a proportional bias, where the difference between the values from the two methods increased or decreased proportionally with the mean values (Figure 1).

### 3.5. Prediction Evaluation Using BIA

The predictive accuracy of BIA for sarcopenia is shown in Table 4. The prediction evaluation using a confusion matrix was calculated as follows:

sensitivity = TP/(TP + FN)(1)

specificity = TN/(TN + FP)(2)

PPV = TP/(TP + FP)(3)

NPV = TN/(TN + FN)(4)

accuracy = (TP + TN)/(TP + FP + FN + TN),(5)

where TP is true positive, TN is true negative, FN is false positive, PPV is positive predictive value, and NPV is negative predictive value. For all participants, the sensitivity, specificity, PPV, NPV, and accuracy were 41.9%, 99.2%, 98.0%, 64.0%, and 71.1%, respectively. In the youth group, these values were 64.0%, 100.0%, 100.0%, 55.0%, and 75.0%, respectively; in the younger group, 36.6%, 97.6%, 93.3%, 58.0%, and 64.3%, respectively; in the middle-aged group, 32.6%, 97.6%, 93.3%, 58.0%, and 64.3%, respectively; and in the elderly group, 50.0%, 100.0%, 100.0%, 71.4%, and 77.8%, respectively.

## 4. Discussion

The reliability, validity, and predictive performance of BIA for assessing sarcopenia were evaluated. While significant differences were observed in the diagnosis of sarcopenia between BIA and DEXA, both methods exhibited a high correlation, fell within the LOA, and demonstrated high predictive accuracy. Therefore, our study indicates the potential utility of these methods as valuable tools for diagnosing sarcopenia.

Prevention and management of sarcopenia are major clinical concerns. Recently, research has been conducted on the diagnostic and treatment methods. Previous studies on BIA and DEXA have mostly compared evaluations based on conditions such as obesity, and there is still a shortage of research specifically focusing on sarcopenia. In this study, we classified BIA reliability and validity for sarcopenia based on crucial factors, such as age and segment. In addition, we evaluated the predictive accuracy for sarcopenia. Various methods, including MRI, CT, DEXA, and BIA, are utilized to estimate skeletal muscle mass. In Asia, DEXA and BIA are commonly used methods. The high cost, time consumption, and radiation exposure associated with CT scans and the inconvenience of community screening have limited the widespread application of CT. In MRI, there is no risk of radiation exposure; however, owing to reasons such as high cost, long examination time, regional disparities in accessibility, and limited compatibility with metal implants, its use as a screening method for elderly individuals is restricted [12,13,21]. DEXA is recognized as a viable alternative method for differentiating fat, bone minerals, and lean tissues. In addition, DEXA is commonly used to measure muscle mass in studies on sarcopenia. However, despite minimal radiation exposure, implementing DEXA in community screening for sarcopenia remains challenging [22]. Therefore, in 2014, the AWGS acknowledged the challenges of measuring muscle mass in community settings and endorsed the use of BIA for ASM measurements [19].

The AWGS considered previous research and provided ASMI cutoff values for sarcopenia diagnosis using BIA (males: <7.0 kg/m^2^; females: <5.7 kg/m^2^) and DEXA (males: <7.0 kg/m^2^; females: <5.4 kg/m^2^). In this study, participants were categorized into low and normal ASM groups based on DEXA measurements. As a result, in the low ASM group, the ASMI for males and females was 7.0 (0.9) kg/m^2^ and 5.7 (0.5) kg/m^2^, respectively, matching the diagnostic criteria set by the AWGS. The calf circumference was used for sarcopenia, with the criteria being <34 cm for males and <33 cm for females. In this study, anthropometric measurements of the arm were obtained at the triceps and those of the leg were obtained at the thigh. Despite the differences in the measurement methods used for assessing sarcopenia, significant differences were observed in the RAC, LAC, WC, HC, RLC, and LLC between the low and normal ASM groups (*p* < 0.05).

In the comparison between DEXA and BIA, FM and %BF were lower than those measured using DEXA (underestimation), whereas FFM, lean mass, ASM, and ASMI were higher (overestimation) (*p* < 0.05). McLester et al. [14] assessed the agreement between three BIA methods (InBody 230, 720, and 770) and DEXA in a study involving 31 males and 36 females. Systematic bias, including underestimation of %BF and FM and overestimation of FFM, was observed in females. A proportional bias was also observed for FM in females and males (*p* < 0.05). In addition, there were significant differences between the DEXA and InBody devices in terms of %BF, FM, and FFM in both males and females; however, a high correlation was observed. Antonio et al. [13] assessed individuals undergoing a 4-week exercise training program and found that BIA (InBody 770) underestimated FM and %BF and overestimated FFM compared with DEXA. Jayanama et al. [23] compared BIA (InBody 720) with DEXA in patients undergoing hemodialysis. They found that the FM, %BF, and FM index were underestimated, whereas FFM was overestimated (*p* < 0.05), and a strong correlation was observed. Yang et al. [15] compared the lean body mass between BIA (ACCUNIQ BC720, BC360, and BC380) and DEXA in 200 healthy adults and found a strong correlation. Therefore, the results of our study are similar to those of previous studies.

In this study, the reliability and validity of BIA were evaluated and compared with those of DEXA by age and segment groups in patients with low and normal ASM. The correlation coefficients (r) of ASMI in the low ASM group were 0.831 for males and 0.822 for females. In the normal ASM group, it was 0.792 and 0.738 for males and females, respectively, indicating a high correlation. Additionally, the r-squared values for the linear regression equations in the RA, LA, RL, and LL were high in the low ASM group, with males having values of 0.914, 0.925, 0.963, and 0.959, respectively, and females with values of 0.791, 0.779, 0.889, and 0.885, respectively (*p* < 0.05). Bland–Altman analysis revealed that in both the low and normal ASM groups, the BIA measurement results for the RA, LA, RL, and LL were within the LOA. However, there were indications of systemic and proportional biases. According to a study by Wingo et al. [12], BIA demonstrated overestimation in lean mass for the RA and LA and underestimation in the RL. Moreover, it showed a correlation (r) of 0.96 or higher. Lee et al. [21] compared the ASM between BIA (InBody 770) and DEXA based on sex, body mass index, age, and BF. In all classifications, BIA showed overestimation (*p* < 0.001), and the intraclass correlation (ICC) demonstrated a high correlation of 0.928 or above. In a study by Buckinx et al. [24], body composition, including appendicular lean mass divided by height squared, was measured using DEXA and BIA (InBody S10). The agreement between BIA and DEXA was high for the upper limbs, with a 95% confidence interval of 0.92 (0.90–0.94) for the LA and 0.87 (0.83–0.90) for the RA. However, when considering sex and the three age groups (18–34, 35–64, and >65 years), the ICC for lean mass in the RL and LL was observed to be low. In a study by Saito et al. [25], muscle mass was evaluated using BIA (InBody 230) and DEXA in 226 elderly patients (≥65 years) hospitalized with heart failure. BIA showed a systemic difference, with lower evaluations of muscle mass in the arms and legs and lower total ASM compared to DEXA. BIA tends to overestimate or underestimate ASMI compared to DEXA. It is crucial to use an adapted formula to obtain an ASM measurement using BIA that is closer to that measured using DEXA [24]. Therefore, the AWGS 2019 does not recommend the use of home-based BIA devices owing to issues with their diagnostic accuracy [19].

The prediction evaluation of sarcopenia based on BIA measurements showed that the sensitivity, specificity, PPV, NPV, and accuracy for all participants were 41.9%, 99.2%, 98.0%, 64.0%, and 71.1%, respectively. In a study by Jeon et al. [26] involving 199 elderly Korean participants aged 70–92 years, BIA (InBody 770, S10) was compared with DEXA to provide equations suitable for the diagnosis of sarcopenia in both standing and supine modes. As a result, the new BIA equations demonstrated a specificity, PPV, and NPV of 85% or more, except for a sensitivity of approximately 60.0%. Saito et al. [25] assessed sarcopenia in 226 individuals using BIA and DEXA. BIA classified 49 (21.7%) individuals as having normal muscle mass and 177 individuals (78.3%) as having low muscle mass, while DEXA classified 106 (46.9%) and 120 (53.1%) individuals, respectively. The prediction of sarcopenia varies depending on the diagnostic perspective. Although sensitivity may be low, if the PPV is high, it allows for a more accurate assessment of sarcopenia. Therefore, finding an appropriate cutoff value for the new equations is crucial, depending on the perspective of sarcopenia diagnosis.

This study had several limitations. First, the results were analyzed based only on healthy Korean participants, and the estimated values may differ owing to individual variations [21,24]. In addition, different results may have been obtained between athletes and sedentary participants [15]. Second, the small sample size in the youth and elderly groups may have resulted in a lack of representativeness of the entire population. Third, electrical conductivity is proportional to the quantity of water and electrolytes present. Adipose tissue, which has lower water content than other tissues, may exhibit lower electrical conductivity, and this tendency increases with higher fat content [15]. Fourth, BIA was measured in the standing position, and the supine position was not considered. Finally, in future studies, clinical research into the diagnosis of sarcopenia will be necessary, taking into account diverse characteristics, such as ethnic groups, ages, sex, body mass index, and medical conditions, beyond those of the participants in this study. Furthermore, the development of equations adapted on the basis of these considerations is essential.

## 5. Conclusions

Sarcopenia research has gained increasing attention, particularly with the establishment of international diagnostic codes. The use of BIA in the diagnosis of sarcopenia is a growing trend owing to its widespread adoption, which offers advantages in terms of cost-effectiveness and accuracy. A comparison between DEXA and BIA showed a strong correlation between BF, %BF, FFM, and lean mass. In both the low and normal ASM groups, the participants exhibited statistically significant differences between BIA and DEXA for each segment (RA, LA, RL, and LL), ASM, and ASMI. However, strong correlations were observed, and the Bland–Altman analysis showed the presence of agreement (LOA) intervals. In addition, high predictive accuracy was demonstrated. Therefore, BIA can be considered a useful and effective tool for diagnosing sarcopenia.

## Figures and Tables

**Table 1:** Characteristics of subjects.

Characteristics	Total			Low ASM			Normal ASM		
	All (*n* = 239)	Male (*n* = 115)	Female (*n* = 124)	All (*n* = 117)	Male (*n* = 46)	Female (*n* = 71)	All (*n* = 122)	Male (*n* = 69)	Female (*n* = 53)
Age (years)	36.2 (16.2)	35.6 (16.1)	36.7 (16.4)	35.1 (17.8)	36.1 (19.8)	34.4 (16.4)	37.2 (14.6)	35.3 (13.2)	39.8 (16.1)
<19	36	15	21	25	10	15	11	5	6
19–39	101	57	44	41	16	25	60	41	19
40–59	84	34	50	43	15	28	41	19	22
≥60	18	9	9	8	5	3	10	4	6
Weight (kg)	64.8 (14.7)	72.4 (15.4) *	57.8 (9.6)	56.8 (11.4)	62.1 (13.8) *	53.4 (7.9)	72.5 (13.3) †	79.2 (12.3) *†	63.8 (8.5) †
Height (cm)	164.1 (11.7)	170.9 (11.9) *	157.9 (7.2)	161.7 (13.0)	167.7 (16.6) *	157.8 (8.1)	166.5 (9.8) †	173.1 (6.6) *	157.9 (5.8)
BMI (kg/m^2^)	23.8 (3.8)	24.5 (4.0) *	23.2 (3.5)	21.5 (2.6)	21.7 (3.0)	21.3 (2.3)	26.1 (3.4) †	26.4 (3.4) †	25.6 (3.3) †
DEXA									
FM (kg)	21.4 (6.4) ‡	20.7 (7.0) ‡	22.0 (5.8) ‡	19.0 (5.2) ‡	17.6 (5.8) ‡	19.9 (4.5) *‡	23.7 (6.7) †‡	22.8 (7.0) †‡	24.8 (6.1) †‡
%BF	33.6 (7.2) ‡	28.7 (5.8) ‡	38.2 (4.9) *‡	34.1 (6.7) ‡	28.7 (6.1) ‡	37.6 (4.3) *‡	33.2 (7.6) ‡	28.7 (5.7) ‡	38.9 (5.6) *‡
FFM (kg)	42.6 (11.1)	50.7 (10.0) *	35.0 (4.9)	37.0 (8.8)	43.6 (9.8) *	32.7 (4.4)	47.9 (10.4) †	55.4 (7.0) *†	38.2 (3.8) †
LM (kg)	40.4 (10.6)	48.2 (9.6) *	33.1 (4.7)	35.0 (8.4)	41.4 (9.2) *	30.8 (4.1)	45.5 (9.9) †	52.7 (6.8) *†	36.2 (3.7) †
BIA									
FM (kg)	18.7 (7.1)	17.3 (7.9)	19.9 (6.2) *	16.1 (5.5)	14.1 (6.1)	17.4 (4.6) *	21.1 (7.7) †	19.5 (8.2) †	23.3 (6.4) *†
%BF	28.8 (8.5)	23.3 (7.6)	33.9 (5.8) *	28.4 (7.8)	22.4 (7.6)	32.3 (4.9) *	29.2 (9.2)	24.0 (7.6)	36.0 (6.2) *†
FFM (kg)	46.1 (11.9) ‡	55.0 (10.7) *‡	37.9 (4.9) ‡	40.7 (9.7) ‡	48.0 (10.8) *‡	35.9 (4.6) ‡	51.4 (11.5) †‡	59.8 (7.7) *†‡	40.5 (4.1) †‡
LM (kg)	43.5 (11.3) ‡	52.0 (10.1) *‡	35.6 (4.6) ‡	38.3 (9.2) ‡	45.3 (10.3) *‡	33.8 (4.4) ‡	48.5 (10.9) †‡	56.4 (7.2) *†‡	38.1 (3.8) †‡
Anthropometric measurements									
RAC (cm)	28.6 (3.9)	30.8 (3.8) *	26.7 (2.9)	26.4 (3.0)	28.0 (3.2) *	25.3 (2.3)	30.8 (3.5) †	32.7 (2.8) *†	28.4 (2.7) †
LAC (cm)	28.2 (3.9)	30.3 (3.7) *	26.3 (2.9)	26.0 (2.9)	27.5 (3.1) *	25.0 (2.2)	30.3 (3.5) †	32.1 (2.8) *†	28.1 (2.8) †
WC (cm)	78.9 (10.6)	82.8 (10.8) *	75.3 (9.0)	73.6 (9.0)	77.2 (10.8) *	71.3 (6.7)	84.0 (9.5) †	86.6 (9.2) *†	80.6 (8.9) †
HC (cm)	95.5 (8.5)	95.8 (10.2)	95.1 (6.6)	91.5 (7.7)	89.8 (9.5)	92.6 (6.1)	99.3 (7.5) †	99.9 (8.6) †	98.5 (5.6) †
RLC (cm)	50.0 (5.6)	50.2 (6.1)	49.8 (5.1)	46.8 (4.7)	45.8 (5.3)	47.5 (4.2)	53.1 (4.5) †	53.2 (4.6) †	52.9 (4.5) †
LLC (cm)	49.8 (5.5)	49.9 (6.0)	49.7 (5.1)	46.6 (4.7)	45.4 (5.3)	47.3 (4.1) *	52.8 (4.5) †	52.8 (4.4) †	52.8 (4.6) †

Characteristics are presented as mean (standard deviation) unless otherwise indicated. ASM: appendicular skeletal muscle mass; BMI: body mass index; DEXA: dual-energy X-ray absorptiometry; BIA: bioelectrical impedance analysis; FM: fat mass; %BF: percentage body fat; FFM: fat-free mass; LM: lean mass; RAC: right arm circumference; LAC: left arm circumference; WC: waist circumference; HC: hip circumference; RLC: right leg circumference; LLC: left leg circumference. * *p* < 0.05, significant difference between males and females. † *p* < 0.05, significant difference between low and normal ASM. ‡ *p* < 0.05, significant difference between DEXA and BIA.

**Table 2:** Comparison of DEXA and BIA in low ASM.

	Total				Male				Female			
	DEXA	BIA	*p*-Value	*r*	DEXA	BIA	*p*-Value	*r*	DEXA	BIA	*p*-Value	*r*
Total (*n* = 117)												
Right arm (kg)	1.7 (0.6)	2.0 (0.7)	<0.001	0.950 *	2.3 (0.6)	2.5 (0.7)	<0.001	0.849 *	1.4 (0.2)	1.6 (0.3)	<0.001	0.849 *
Left arm (kg)	1.6 (0.6)	1.9 (0.7)	<0.001	0.948 *	2.1 (0.6)	2.5 (0.7)	<0.001	0.845 *	1.3 (0.2)	1.6 (0.3)	<0.001	0.850 *
Right leg (kg)	5.5 (1.5)	6.3 (1.7)	<0.001	0.969 *	6.7 (1.6)	7.6 (1.8)	<0.001	0.954 *	4.7 (0.7)	5.5 (0.8)	<0.001	0.899 *
Left leg (kg)	5.5 (1.5)	6.3 (1.6)	<0.001	0.968 *	6.7 (1.6)	7.6 (1.8)	<0.001	0.920 *	4.7 (0.7)	5.5 (0.8)	<0.001	0.905 *
ASM (kg)	14.3 (4.1)	16.6 (4.6)	<0.001	0.977 *	17.8 (4.2)	20.2 (5.0)	<0.001	0.934 *	12.1 (1.7)	14.2 (2.2)	<0.001	0.930 *
ASMI (kg/m^2^)	5.3 (0.9)	6.2 (1.0)	<0.001	0.936 *	6.2 (0.8)	7.0 (0.9)	<0.001	0.831 *	4.8 (0.4)	5.7 (0.5)	<0.001	0.822 *
Youth group (*n* = 25)												
Right arm (kg)	1.3 (0.5)	1.5 (0.7)	<0.001	0.975 *	1.5 (0.7)	1.6 (1.0)	0.103	0.994 *	1.2 (0.3)	1.4 (0.5)	<0.001	0.937 *
Left arm (kg)	1.2 (0.5)	1.5 (0.7)	<0.001	0.970 *	1.4 (0.7)	1.6 (1.0)	0.052	0.995 *	1.1 (0.3)	1.4 (0.4)	<0.001	0.914 *
Right leg (kg)	4.7 (1.7)	5.5 (1.9)	<0.001	0.991 *	5.0 (2.4)	5.7 (2.7)	<0.001	0.993 *	4.5 (1.1)	5.3 (1.3)	<0.001	0.987 *
Left leg (kg)	4.7 (1.7)	5.4 (1.9)	<0.001	0.992 *	4.9 (2.4)	5.6 (2.6)	<0.05	0.976 *	4.5 (1.1)	5.2 (1.3)	<0.001	0.971 *
ASM (kg)	11.9 (4.4)	13.9 (5.2)	<0.001	0.991 *	12.8 (6.1)	14.6 (7.3)	<0.05	0.976 *	11.4 (2.8)	13.4 (3.4)	<0.001	0.964 *
ASMI (kg/m^2^)	4.9 (0.9)	5.6 (1.0)	<0.001	0.925 *	5.2 (1.1)	5.9 (1.3)	<0.05	0.939 *	4.6 (0.5)	5.5 (0.7)	<0.001	0.850 *
Younger group (*n* = 41)												
Right arm (kg)	1.8 (0.7)	2.0 (0.7)	<0.001	0.957 *	2.6 (0.3)	2.8 (0.4)	<0.05	0.762 *	1.4 (0.2)	1.6 (0.3)	<0.001	0.885 *
Left arm (kg)	1.7 (0.6)	2.1 (0.7)	<0.001	0.962 *	2.4 (0.3)	2.8 (0.4)	<0.05	0.747 *	1.3 (0.2)	1.6 (0.3)	<0.001	0.928 *
Right leg (kg)	5.9 (1.4)	6.8 (1.7)	<0.001	0.962 *	7.4 (0.9)	8.6 (1.2)	<0.001	0.968 *	4.9 (0.6)	5.7 (0.6)	<0.001	0.851 *
Left leg (kg)	5.8 (1.5)	6.7 (1.7)	<0.001	0.961 *	7.4 (0.9)	8.5 (1.2)	<0.001	0.950 *	4.8 (0.5)	5.6 (0.6)	<0.001	0.859 *
ASM (kg)	15.3 (4.1)	17.7 (4.7)	<0.001	0.976 *	19.9 (2.3)	22.7 (3.2)	<0.001	0.948 *	12.3 (1.4)	14.4 (1.8)	<0.001	0.920 *
ASMI (kg/m^2^)	5.4 (0.9)	6.3 (1.0)	<0.001	0.931 *	6.4 (0.4)	7.3 (0.6)	<0.001	0.788 *	4.8 (0.4)	5.7 (0.5)	<0.001	0.819 *
Middle-aged group (*n* = 43)												
Right arm (kg)	1.8 (0.6)	2.1 (0.6)	<0.001	0.925 *	2.6 (0.2)	2.8 (0.3)	<0.001	0.665 *	1.4 (0.2)	1.7 (0.3)	<0.001	0.782 *
Left arm (kg)	1.7 (0.5)	2.0 (0.6)	<0.001	0.922 *	2.3 (0.2)	2.7 (0.3)	<0.001	0.610 *	1.3 (0.2)	1.7 (0.3)	<0.001	0.777 *
Right leg (kg)	5.5 (1.2)	6.4 (1.4)	<0.001	0.963 *	7.0 (0.7)	8.0 (0.8)	<0.001	0.799 *	4.7 (0.5)	5.5 (0.6)	<0.001	0.907 *
Left leg (kg)	5.6 (1.2)	6.4 (1.4)	<0.001	0.954 *	7.1 (0.7)	8.0 (0.8)	<0.001	0.781 *	4.8 (0.5)	5.5 (0.6)	<0.001	0.882 *
ASM (kg)	14.6 (3.5)	16.9 (3.9)	<0.001	0.959 *	18.9 (1.7)	21.5 (2.1)	<0.001	0.779 *	12.3 (1.2)	14.5 (1.8)	<0.001	0.892 *
ASMI (kg/m^2^)	5.5 (0.8)	6.3 (0.9)	<0.001	0.915 *	6.5 (0.4)	7.3 (0.4)	<0.001	0.554 *	4.9 (0.4)	5.8 (0.5)	<0.001	0.787 *
Elderly group (*n* = 8)												
Right arm (kg)	1.9 (0.4)	2.3 (0.5)	<0.001	0.988 *	2.2 (0.2)	2.6 (0.3)	0.001	0.942 *	1.5 (0.2)	1.7 (0.2)	<0.05	0.923
Left arm (kg)	1.8 (0.5)	2.3 (0.5)	<0.001	0.987 *	2.2 (0.2)	2.6 (0.2)	<0.001	0.870	1.3 (0.2)	1.7 (0.3)	<0.05	0.996
Right leg (kg)	5.8 (1.2)	6.5 (1.3)	<0.001	0.980 *	6.6 (0.5)	7.3 (0.7)	<0.05	0.956 *	4.5 (0.4)	5.1 (0.4)	0.053	0.845
Left leg (kg)	5.9 (1.4)	6.5 (1.3)	<0.05	0.977 *	6.8 (0.6)	7.3 (0.7)	<0.05	0.860	4.3 (0.4)	5.0 (0.4)	<0.05	0.995
ASM (kg)	15.5 (3.4)	17.5 (3.6)	<0.001	0.986 *	17.8 (1.4)	19.9 (1.8)	<0.05	0.912 *	11.6 (1.1)	13.5 (1.2)	<0.05	0.954
ASMI (kg/m^2^)	5.9 (1.0)	6.6 (0.9)	<0.001	0.975 *	6.5 (0.3)	7.3 (0.4)	<0.05	0.705	4.7 (0.1)	5.5 (0.2)	<0.05	0.462

Characteristics are expressed as mean (standard deviation). DEXA, dual-energy X-ray absorptiometry; BIA, bioelectrical impedance analysis; ASM, appendicular skeletal muscle mass; ASMI, appendicular skeletal muscle mass index. * *p* < 0.05, correlation coefficient between DEXA and BIA.

**Table 3:** Comparison of DEXA and BIA in normal ASM.

	Total				Male				Female			
	DEXA	BIA	*p*-Value	*r*	DEXA	BIA	*p*-Value	*r*	DEXA	BIA	*p*-Value	*r*
Total (*n* = 122)												
Right arm (kg)	2.5 (0.9)	2.7 (0.8)	<0.001	0.949 *	3.2 (0.5)	3.4 (0.5)	<0.001	0.812 *	1.7 (0.3)	1.9 (0.3)	<0.001	0.803 *
Left arm (kg)	2.3 (0.8)	2.7 (0.8)	<0.001	0.933 *	2.9 (0.4)	3.3 (0.5)	<0.001	0.746 *	1.5 (0.2)	1.9 (0.3)	<0.001	0.759 *
Right leg (kg)	7.5 (1.8)	8.2 (2.1)	<0.001	0.974 *	8.8 (1.3)	9.7 (1.4)	<0.001	0.920 *	5.8 (0.7)	6.3 (0.8)	<0.001	0.883 *
Left leg (kg)	7.5 (1.8)	8.2 (2.0)	<0.001	0.980 *	8.8 (1.3)	9.6 (1.4)	<0.001	0.937 *	5.8 (0.7)	6.2 (0.8)	<0.001	0.912 *
ASM (kg)	19.8 (5.1)	21.9 (5.7)	<0.001	0.980 *	23.6 (3.3)	26.1 (3.7)	<0.001	0.927 *	14.8 (1.7)	16.4 (2.0)	<0.001	0.923 *
ASMI (kg/m^2^)	7.0 (1.2)	7.7 (1.3)	<0.001	0.937 *	7.9 (0.8)	8.7 (0.9)	<0.001	0.792 *	5.9 (0.5)	6.5 (0.6)	<0.001	0.738 *
Youth group (*n* = 11)												
Right arm (kg)	2.4 (1.2)	2.6 (1.2)	<0.05	0.948 *	3.3 (1.2)	3.6 (1.2)	0.063	0.821	1.6 (0.1)	1.8 (0.2)	<0.05	0.771
Left arm (kg)	2.0 (0.8)	2.6 (1.1)	<0.05	0.970 *	2.8 (0.4)	3.5 (1.1)	0.063	1.000 *	1.4 (0.1)	1.8 (0.2)	<0.05	0.812
Right leg (kg)	7.6 (2.5)	8.4 (2.7)	<0.05	0.964 *	9.6 (2.5)	10.7 (2.6)	0.063	0.700	6.0 (0.4)	6.6 (0.3)	<0.05	0.943 *
Left leg (kg)	7.7 (2.5)	8.3 (2.7)	<0.05	0.955 *	9.7 (2.7)	10.5 (2.6)	0.063	0.900	6.1 (0.3)	6.5 (0.3)	<0.05	0.771
ASM (kg)	19.8 (6.9)	22.0 (7.6)	<0.05	0.973 *	25.4 (6.7)	28.2 (7.4)	0.063	0.800	15.1 (0.9)	16.8 (1.0)	<0.05	0.943 *
ASMI (kg/m^2^)	7.0 (1.8)	7.7 (2.0)	<0.05	0.891 *	8.4 (1.9)	9.3 (2.1)	0.063	1.000 *	5.8 (0.3)	6.4 (0.2)	<0.05	0.314
Younger group (*n* = 60)												
Right arm (kg)	2.7 (0.9)	2.9 (0.8)	<0.001	0.925 *	3.2 (0.5)	3.4 (0.5)	<0.05	0.795 *	1.6 (0.2)	1.9 (0.2)	<0.001	0.692 *
Left arm (kg)	2.5 (0.8)	2.9 (0.8)	<0.001	0.893 *	3.0 (0.5)	3.4 (0.5)	<0.001	0.702 *	1.5 (0.2)	1.9 (0.2)	<0.001	0.630 *
Right leg (kg)	8.0 (1.8)	8.9 (2.0)	<0.001	0.971 *	9.0 (1.1)	10.0 (1.2)	<0.001	0.907 *	5.9 (0.6)	6.4 (0.7)	<0.001	0.842 *
Left leg (kg)	8.1 (1.7)	8.8 (2.0)	<0.001	0.975 *	9.1 (1.0)	9.9 (1.2)	<0.001	0.916 *	5.9 (0.6)	6.4 (0.7)	<0.001	0.864 *
ASM (kg)	21.3 (5.1)	23.5 (5.6)	<0.001	0.973 *	24.3 (2.9)	26.8 (3.3)	<0.001	0.924 *	14.9 (1.5)	16.5 (1.8)	<0.001	0.900 *
ASMI (kg/m^2^)	7.3 (1.2)	8.1 (1.3)	<0.001	0.951 *	8.0 (0.8)	8.8 (0.8)	<0.001	0.863 *	5.9 (0.4)	6.5 (0.4)	<0.001	0.731 *
Middle-aged group (*n* = 41)												
Right arm (kg)	2.3 (0.7)	2.6 (0.7)	<0.001	0.946 *	3.0 (0.3)	3.2 (0.4)	<0.001	0.712 *	1.8 (0.3)	2.0 (0.3)	<0.001	0.847 *
Left arm (kg)	2.2 (0.6)	2.5 (0.7)	<0.001	0.954 *	2.8 (0.2)	3.2 (0.4)	<0.001	0.753 *	1.6 (0.3)	2.0 (0.3)	<0.001	0.879 *
Right leg (kg)	6.8 (1.5)	7.5 (1.7)	<0.001	0.963 *	8.1 (1.0)	9.0 (1.1)	<0.001	0.940 *	5.7 (0.7)	6.2 (0.8)	<0.001	0.828 *
Left leg (kg)	6.8 (1.5)	7.5 (1.7)	<0.001	0.967 *	8.1 (1.0)	9.0 (1.1)	<0.001	0.904 *	5.7 (0.7)	6.2 (0.8)	<0.001	0.874 *
ASM (kg)	18.1 (4.2)	20.1 (4.8)	<0.001	0.971 *	22.0 (2.4)	24.5 (2.9)	<0.001	0.921 *	14.8 (1.9)	16.3 (2.2)	<0.001	0.873 *
ASMI (kg/m^2^)	6.7 (1.0)	7.5 (1.1)	<0.001	0.895 *	7.6 (0.4)	8.4 (0.5)	<0.001	0.607 *	6.0 (0.6)	6.6 (0.7)	<0.001	0.671 *
Elderly group (*n* = 10)												
Right arm (kg)	2.2 (0.6)	2.4 (0.6)	<0.001	0.979 *	2.8 (0.3)	3.1 (0.3)	<0.05	0.977 *	1.8 (0.3)	2.0 (0.4)	<0.05	0.956 *
Left arm (kg)	2.0 (0.6)	2.4 (0.7)	<0.001	0.978 *	2.6 (0.3)	3.1 (0.3)	<0.05	0.922	1.6 (0.2)	1.9 (0.4)	<0.05	0.920 *
Right leg (kg)	6.5 (1.6)	7.0 (1.7)	<0.001	0.990 *	8.1 (0.7)	8.7 (0.9)	<0.05	0.998 *	5.4 (0.8)	5.8 (1.0)	<0.05	0.962 *
Left leg (kg)	6.5 (1.6)	7.0 (1.7)	<0.001	0.993 *	8.1 (0.7)	8.6 (0.9)	<0.05	0.979 *	5.5 (0.9)	5.8 (1.0)	<0.05	0.980 *
ASM (kg)	17.3 (4.3)	18.8 (4.8)	<0.001	0.995 *	21.7 (2.0)	23.5 (2.4)	<0.05	0.992 *	14.3 (2.2)	15.6 (2.8)	<0.05	0.984 *
ASMI (kg/m^2^)	6.6 (0.9)	7.2 (1.0)	<0.001	0.980 *	7.4 (0.3)	8.1 (0.5)	<0.05	0.987 *	6.0 (0.5)	6.6 (0.8)	<0.05	0.966 *

Characteristics are expressed as mean (standard deviation). DEXA, dual-energy X-ray absorptiometry; BIA, bioelectrical impedance analysis; ASM, appendicular skeletal muscle mass; ASMI, appendicular skeletal muscle mass index. * *p* < 0.05, correlation coefficient between DEXA and BIA.

**Table 4:** Predictions of sarcopenia using BIA.

	TP	FP	FN	TN	Sen (%)	Spec (%)	PPV (%)	NPV (%)	Acc (%)
Total	49	1	68	121	41.9	99.2	98.0	64.0	71.1
Male	15	0	31	69	32.6	100.0	100.0	69.0	73.0
Female	34	1	37	52	47.9	98.1	97.1	58.4	69.4
Youth group	16	0	9	11	64.0	100.0	100.0	55.0	75.0
Male	8	0	2	5	80.0	100.0	100.0	71.4	86.7
Female	8	0	7	6	53.3	100.0	100.0	46.2	66.7
Younger group	15	0	26	60	36.6	100.0	100.0	69.8	74.3
Male	4	0	12	41	25.0	100.0	100.0	77.4	78.9
Female	11	0	14	19	44.0	100.0	100.0	57.6	68.2
Middle-aged group	14	1	29	40	32.6	97.6	93.3	58.0	64.3
Male	2	0	13	19	13.3	100.0	100.0	59.4	61.8
Female	12	1	16	21	42.9	95.5	92.3	56.8	66.0
Elderly group	4	0	4	10	50.0	100.0	100.0	71.4	77.8
Male	1	0	4	4	20.0	100.0	100.0	50.0	55.6
Female	3	0	0	6	100.0	100.0	100.0	100.0	100.0

Characteristics are expressed as the mean (SD) of the number of risk factors. TP, true positive; FP, false positive; FN, false negative; TN, true negative; Sen, sensitivity; Spec, specificity; PPV, positive predictive value; NPV, negative predictive value; Acc, accuracy.

## Data Availability

Data can be provided upon request owing to restrictions: The data presented in this study are available on request from the corresponding author. The data are not publicly available due to privacy.
